# Supplementary material for: Rat donor lung quality deteriorates more after fast than slow brain death induction
Source: PLoS One. 2020 Nov 30;15(11):e0242827. doi: 10.1371/journal.pone.0242827 (PMC7704005; doi:10.1371/journal.pone.0242827)
Supplement: S1 File — (PDF) [file pone.0242827.s003.pdf]

Rebolledo et al. J Transl Med (2016) 14:141
DOI 10.1186/s12967-016-0890-0

Journal of Translational Medicine

RESEARCH Open Access

Slow induction of brain death leads to decreased renal function and increased hepatic apoptosis in rats

Rolando A. Rebolledo[1,2*†], Dane Hoeksma[1†], Christina M. V. Hottenrott[3], Yves J. L. Bodar[1], Petra J. Ottens[1], Janneka Wiersema-Buist[1] and Henri G. D. Leuvenink[1]

Abstract

Background: Donor brain death (BD) is an independent risk factor for graft survival in recipients. While in some patients BD results from a fast increase in intracranial pressure, usually associated with trauma, in others, intracranial pressure increases more slowly. The speed of intracranial pressure increase may be a possible risk factor for renal and hepatic graft dysfunction. This study aims to assess the effect of speed of BD induction on renal and hepatic injury markers.

Methods: BD induction was performed in 64 mechanically ventilated male Fisher rats by inflating a 4.0F Fogarty catheter in the epidural space. Rats were observed for 0.5, 1, 2 or 4 h following BD induction. Slow induction was achieved by inflating the balloon-catheter at a speed of 0.015 ml/min until confirmation of BD. Fast induction was achieved by inflating the balloon at 0.45 ml/min for 1 min. Plasma, kidney and liver tissue were collected for analysis.

Results: Slow BD induction led to higher plasma creatinine at all time points compared to fast induction. Furthermore, slow induction led to increased renal mRNA expression of IL-6, and renal MDA values after 4 h of BD compared to fast induction. Hepatic mRNA expression of TNF-α, Bax/Bcl-2, and protein expression of caspase-3 was significantly higher due to slow induction after 4 h of BD compared to fast induction. PMN infiltration was not different between fast and slow induction in both renal and hepatic tissue.

Conclusion: Slow induction of BD leads to poorer renal function compared to fast induction. Renal inflammatory and oxidative stress markers were increased. Liver function was not affected by speed of BD induction but hepatic inflammatory and apoptosis markers increased significantly due to slow induction compared to fast induction. These results provide initial proof that speed of BD induction influences detrimental renal and hepatic processes which could signify different donor management strategies for patients progressing to BD at different speeds.

Keywords: Brain death, Organ donation, Kidney transplantation, Liver transplantation

Background

The shortage of qualitative donor organs remains a limiting factor in organ transplantation. Therefore, resorting to sub-optimal donor types to meet the increasing demand of organs is inevitable. Today, brain-dead donors form the largest donor pool worldwide for kidney and liver transplantation [1, 2]. Unfortunately, transplanting kidneys from brain dead donors leads to higher incidences of rejection and delayed graft function compared to living donors [3]. A cerebrovascular cause of BD is related to renal and liver graft failure indicating that the nature of brain insults affects graft function as well [4, 5].

Brain death (BD) is a complex pathological condition, characterized by hemodynamic imbalance, hormonal impairment, and a systemic inflammatory response. Hemodynamic imbalance comprises changes elicited by

*Correspondence: rareboll@uc.cl
†Rolando A. Rebolledo and D. Hoeksma contributed equally to this work
[1] Department of Surgery, University Medical Center Groningen, University of Groningen, CMC V, Y2144, Hanzeplein 1, 9713 GZ, Groningen, The Netherlands
Full list of author information is available at the end of the article

BioMed Central

© 2016 The Author(s). This article is distributed under the terms of the Creative Commons Attribution 4.0 International License (http://creativecommons.org/licenses/by/4.0/), which permits unrestricted use, distribution, and reproduction in any medium, provided you give appropriate credit to the original author(s) and the source, provide a link to the Creative Commons license, and indicate if changes were made. The Creative Commons Public Domain Dedication waiver (http://creativecommons.org/publicdomain/zero/1.0/) applies to the data made available in this article, unless otherwise stated.

brainstem herniation, the resulting catecholamine storm, and neurogenic shock due to ischemia of the spinal cord. Systemic inflammation is characterised by increased levels of circulating cytokines including interleukin-6 (IL-6), interleukin-10 (IL-10), tumor necrosis factor-alpha (TNF-α), transforming growth factor-beta (TGF-β) and, monocyte chemotactic protein 1 (MCP-1) [6–8]. This systemic inflammatory environment promotes the migration of inflammatory cells into organs triggering a local inflammatory and (pro-)apoptotic response [9, 10]. Furthermore, BD affects pituitary function causing a decrease in plasma levels of cortisol, thyroid hormones (T_3/T_4), insulin, and antidiuretic hormone (ADH) [11–13].

Brain death-related processes such as the catecholamine storm are affected by the speed at which intracranial pressure (ICP) increases. A faster increase in ICP leads to higher levels of circulating catecholamines which is detrimental for cardiac and pulmonary graft function [14]. Indeed, traumatic brain injury, the most common cause of BD preceded by a rapid increase in ICP, is a risk factor for mortality in heart recipients [15]. In contrast, a cerebrovascular cause of death, usually preceded by a slower increase in ICP, is a risk factor for renal and hepatic graft dysfunction. However, this phenomenon is not believed to be associated with a slower increase in ICP. Rather, donor characteristics such as obesity, older age, and the presence of cardiovascular disease are regarded as the underlying cause [5, 16, 17].

We aimed to assess whether the speed of BD induction affects renal and hepatic function in brain dead donor rats.

Methods

Animals

Sixty-four male Fisher F344 rats (270–300 g) were subjected to either fast or slow BD induction with a BD duration of 0.5, 1, 2 or 4 h. All animals received care in compliance with the guidelines of the local animal ethics committee according to the Experiments on Animals Act (1996) issued by the Ministry of Public Health, Welfare and Sports of the Netherlands. Animals were anaesthetized using 2–5 % isoflurane with 100 % oxygen. Two millilitre saline was administered s.c. to prevent dehydration. Animals were intubated via a tracheostomy and ventilated (tidal volume: 6.5 ml/kg of body weight, PEEP of 3 cm of H_2O at an initial respiratory rate of 120 and was adjusted to maintain the $ETCO_2$ in normocapnia range) throughout the experiment. Cannulas were inserted in the femoral artery and vein for continuous mean arterial pressure (MAP) monitoring and volume replacement. Through a frontolateral hole drilled in the skull, a no. 4 Fogarty catheter (Edwards Lifesciences Co, Irvine, CA) was placed in the epidural space and inflated with saline using a syringe pump (Terufusion, Termo Co., Tokyo, Japan). Fast and slow induction of BD were induced by inflating the catheter at a speed of 0.45 or 0.015 ml/min, respectively. For slow speed induction, inflation of the balloon was terminated when the MAP increased above 80 mmHg due to the catecholamine storm. For fast induction, the catheter was inflated over a period of 1 min. BD was confirmed by the absence of corneal reflexes half an hour after induction after which anaesthesia was discontinued. MAP was maintained above 80 mmHg. If necessary, colloid infusion with 10 % polyhydroxyethyl starch (HAES) (Fresenius Kabi AG, Bad Homburg, Germany) was given in a bolus (limited to a maximum of 1 ml/h) to maintain the MAP above 80 mmHg. Unresponsiveness to HAES indicated the start of an intravenous noradrenaline (NA) drip (1 mg/ml). A homeothermic blanket control system was used throughout the experiment, maintaining the body temperature between 37 and 38 °C. At the end of the experimental period, blood and urine were collected, and the animals were systemically flushed with cold saline. After the flush, organs were harvested and tissue samples were snap frozen in liquid nitrogen and stored at −80 °C or fixated in 4 % paraformaldehyde. Plasma samples and urine were also snap-frozen and stored. One animal was discarded in the slow induction 2 h group, two animals in the fast induction group 2 h and one in the fast induction 4 h group due to unknown amounts of noradrenaline administration. One animal was discarded in the fast induction 4 h group due to an apnea test conducted during the BD period.

Animals were randomly assigned to one of 8 experimental groups (n = 8):

- Fast BD induction 0.5 h
- Fast BD induction 1 h
- Fast BD induction 2 h
- Fast BD induction 4 h
- Slow BD induction 0.5 h
- Slow BD induction 1 h
- Slow BD induction 2 h
- Slow BD induction 4 h

Biochemical determinations

Plasma levels of alanine transaminase (ALT), aspartate transaminase (AST) and creatinine were determined at the clinical chemistry lab of University Medical Centre Groningen according to standard procedures.

Plasma IL-6 measurement

Plasma IL-6 was determined by a rat enzyme-linked immunosorbent assay (IL-6 ELISA) kit (R&D Systems Europe Ltd. Abingdon, Oxon OX14 3NB, UK), according

to the manufacturer's instructions. All samples were analyzed in duplicate and read at 450 nm.

RNA isolation and cDNA synthesis

Total RNA was isolated from whole liver and kidney sections using TRIzol (Life Technologies, Gaithersburg, MD). Samples were verified for absence of genomic DNA contamination by performing RT-PCR reactions in which the addition of reverse transcriptase was omitted, using Glyceraldehyde 3-phosphate dehydrogenase (GAPDH) primers. For cDNA synthesis, 1 μl T11VN Oligo-dT (0.5 μg/μl) and 1 μg mRNA were incubated for 10 min at 70 °C and cooled directly after that. cDNA was synthesized by adding a mixture containing 0.5 μl RnaseOUT® Ribonuclease inhibitor (Invitrogen, Carlsbad, USA), 0.5 μl RNase water (Promega), 4 μl 5× first strand buffer (Invitrogen), 2 μl DTT (Invitrogen), 1 μl dNTP's and 1 μl M-MLV reverse transcriptase (Invitrogen, 200 U). The mixture was held at 37 °C for 50 min. Next, reverse transcriptase was inactivated by incubating the mixture for 15 min at 70 °C. Samples were stored at −20 °C.

Real-time PCR

Fragments of several genes were amplified with the primer sets outlined in Table 1. Pooled cDNA obtained from brain-dead rats was used as an internal reference. Gene expression was normalized with the mean of β-actin mRNA content. Real-Time PCR was carried out in reaction volumes of 15 μl containing 10 μl of SYBR Green mastermix (Applied biosystems, Foster City, USA), 0.4 μl of each primer (50 μM), 4.2 μl of nuclease free water and 10 ng of cDNA. All samples were analyzed in triplicate. Thermal cycling was performed on the Taqman Applied Biosystems 7900HT Real Time PCR System with a hot start for 2 min at 50 °C followed by 10 min 95 °C. Second stage was started with 15 s at 95 °C (denaturation step) and 60 s at 60 °C (annealing step and DNA synthesis). The latter stage was repeated 40 times. Stage 3 was included to detect formation of primer dimers (melting curve) and begins with 15 s at 95 °C followed by 60 s at 60 °C and 15 s at 95 °C. Primers were designed with Primer Express software (Applied Biosystems) and primer efficiencies were tested by a standard curve for the primer pair resulting from the amplification of serially diluted cDNA samples (10, 5, 2.5, 1.25 and 0.625 ng) obtained from brain-dead rats. PCR efficiency was $1.8 < \varepsilon < 2.0$. Real-time PCR products were checked for product specificity on a 1.5 % agarose gel. Results were expressed as $2^{-\triangle\triangle CT}$ (CT: threshold cycle).

Table 1 Primer sequences used for real-time PCR

Gene	Primers	Amplication size (bp)
IL-6	5′-CCAACTTCCAATGCTCTCCTAATG-3′<br>5′- TTCAAGTGCTTTCAAGAGTTGGAT-3′	89
TNF-α	5′-GGCTGCCTTGGTTCAGATGT-3′<br>5′-CAGGTGGGAGCAACCTACAGTT-3′	79
BAX	5′-GCGTGGTTGCCCTCTTCTAC-3′<br>5′-TGATCAGCTCGGGCACTTTAGT-3′	74
Bcl2	5′-CTGGGATGCCTTTGTGGAA-3′<br>5′-TCAGAGACAGCCAGGAGAAATCA-3′	70

HIS 48 immunological staining

Five-micrometer renal and hepatic cryostat sections were fixed in acetone and stained according to standard protocol. In brief, mouse monoclonal anti-rat granulocyte antibody (HIS 48; IQ products, Groningen) was dissolved in PBS (pH 7.4) supplemented with 1 % bovine serum albumin (BSA). Peroxidase-labeled second step antibody (rabbit anti-mouse) was diluted in 1 % BSA/PBS containing 5 % normal rat serum. Peroxidase activity was visualized using aminoethylcarbazole. Sections were counterstained with Mayers Hemotoxylin. Control sections were incubated with omission of the primary antibody. For each tissue section, positive cells per field were counted by a blinded researcher in ten microscopic fields of the tissue at 20× and 10× magnification for the kidney and liver respectively. Results were presented as number of positive cells per field.

Tissue MDA

Kidney and liver tissue was homogenized with a pestle and mortar in PBS containing 5 mM butylated hydroxytoluene. Malondialdehyde (MDA) was measured fluorescently after binding to thiobarbituric acid. Hundred microlitre of tissue homogenate was mixed with 2 % SDS followed by 400 μl 0.1 N HCL, 50 μl 10 % phosphotungstic acid and 200 μl 0.7 % TBA. The mixture was incubated for 30 min at 97 °C. Eight hundred microlitre 1-butanol was added to the samples and centrifuged at 960g. Two hundred microlitre of the 1-butanol supernatant was fluorescently measured at 480 nm excitation and 590 nm emission wavelengths. Samples were corrected for total amount of protein.

Cleaved caspase-3 staining

To detect caspase-3 positive cells in liver and kidney, immunohistochemistry was performed on 3 μm sections of paraffin embedded liver samples. Sections were deparaffined in a sequence of xylene, alcohol and water. As an antigen retrieval method we used for caspase-3 samples: EDTA (1 mM, pH 8.0) buffer. Next, sections were stained with Caspase-3 primary Antibody (Cell Signaling cat. nr. 9661, 100× diluted in 1 % BSA/PBS) using an indirect immunoperoxidase technique. Endogenous peroxidase

was blocked using H_2O_2 0.3 % in phosphate-buffered saline for 30 min. After thorough washing, sections were incubated with horseradish peroxidase-conjugated goat anti-rabbit IgG as a secondary antibody for 30 min (Dako, Glostrup, Denmark. cat. nr. P0448), followed by rabbit anti-goat IgG as a tertiary antibody for 30 min (Dako, Glostrup, Denmark. cat. nr. P0449). The reaction was developed using DAB as chromogen and H_2O_2 as substrate. Sections were counterstained using Mayer hematoxylin solution (Merck, Darmstadt, Germany). Negative antibody controls were performed. Localization of immunohistochemical staining was assessed by light microscopy. For each tissue section, positive cells per field were counted by a blinded researcher in ten microscopic fields of the tissue at 10× magnification. Results were presented as number of positive cells per field.

Statistical analyses

Statistical analysis was performed between both experimental groups using a nonparametric test (Mann–Whitney) for every time point. All statistical tests were 2-tailed and $p < 0.05$ was regarded as significant. Results are presented as mean ± SD (standard deviation).

Results

As an internal control we compared the catheter volume after brain death induction and blood pressure pattern during the induction phase. The final catheter volume was similar between the slow and fast induction model (0.41 ± 0.03 ml vs 0.41 ± 0.02). During BD induction, the MAP showed different characteristic patterns due to fast and slow speed induction (Fig. 1). Slow speed BD induction was characterized by a period of decreased blood pressure which typically started 10 min before the end of the induction and in which the minimum pressure registered was 51.17 ± 10.76 mmHg. In contrast, fast speed induction was characterized by a sudden and short increase in MAP which was typically observed at the end of the balloon inflation period and in which the maximum pressure registered was 167.39 ± 37.85 mmHg.

The amount of HAES needed for a stable MAP was similar after fast and slow speed induction. The amount of administered NA was significantly higher in the fast induction group compared to slow induction after 0.5 and 1 h of BD (Table 2). We estimated the chance of noradrenaline and HAES utilization using hazard curves. Slow induction led to a 17.05 % probability of NA use in the first hour of BD, while fast induction led to a 54.84 % probability. Additionally, we compared both curves using the Mantel–Cox test. The curves for NA use were significantly different ($p = 0.0004$). HAES was used mainly in the first minutes after BD induction. Slow induction led to a 48.39 % probability of HAES use in the first 10 min of BD while fast induction led to a 84.38 % probability. Curve comparison was found to be significantly different using the Mantel–Cox test ($p = 0.0091$, Fig. 2).

ALT and AST plasma levels were measured as liver cell injury markers. No differences were found in ALT levels between fast and slow speed induction. The AST plasma levels were increased due to fast induction compared to slow induction groups after 0.5 and 2 h of BD ($p = 0.0225$ and $p = 0.0088$, Fig. 3).

Plasma creatinine levels were measured in order to estimate kidney function. Creatinine was significantly higher after slow induction compared to fast induction at every time point. Plasma urea levels were increased due to slow induction compared to fast induction after 4 h of BD ($p = 0.0308$, Fig. 3).

Plasma IL-6 levels were measured as a marker for systemic inflammation. IL-6 plasma levels were significantly

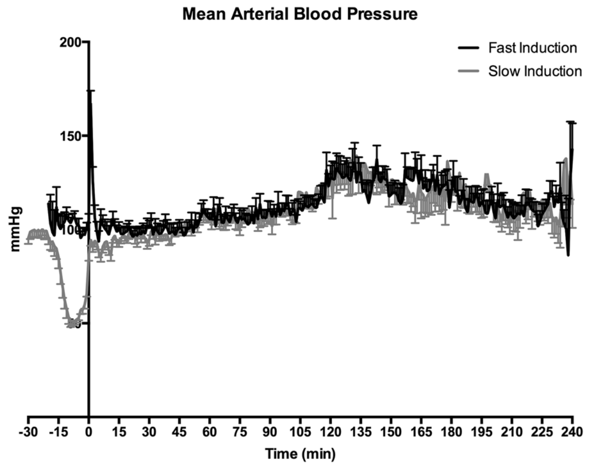

Fig. 1 Course of MAP during BD induction and during 4-h BD in fast- ($n = 32$) and slow- ($n = 35$) inducted rats. $T = 0$ represents the start of the BD period

Table 2 Total noradrenaline (1 mg/ml) and HAES 10 % infusion requirements

	Time (h)	Fast induction	Slow induction	p value
Noradrenaline (ml)	0.5	0.35 ± 0.42	0.10 ± 0.24	0.0188*
	1	0.55 ± 0.76	0.05 ± 0.14	0.0238*
	2	1.1 ± 1.6	0.13 ± 0.25	0.1515
	4	0.33 ± 0.58	0.23 ± 0.42	0.8564
HAES 10 % (ml)	0.5	0.44 ± 0.18	0.31 ± 0.26	0.5692
	1	0.56 ± 0.18	0.50 ± 0.0	0.9999
	2	0.50 ± 0.0	0.38 ± 0.35	0.2000
	4	0.56 ± 0.50	0.56 ± 0.42	0.9999

Asterisk indicates $p < 0.05$

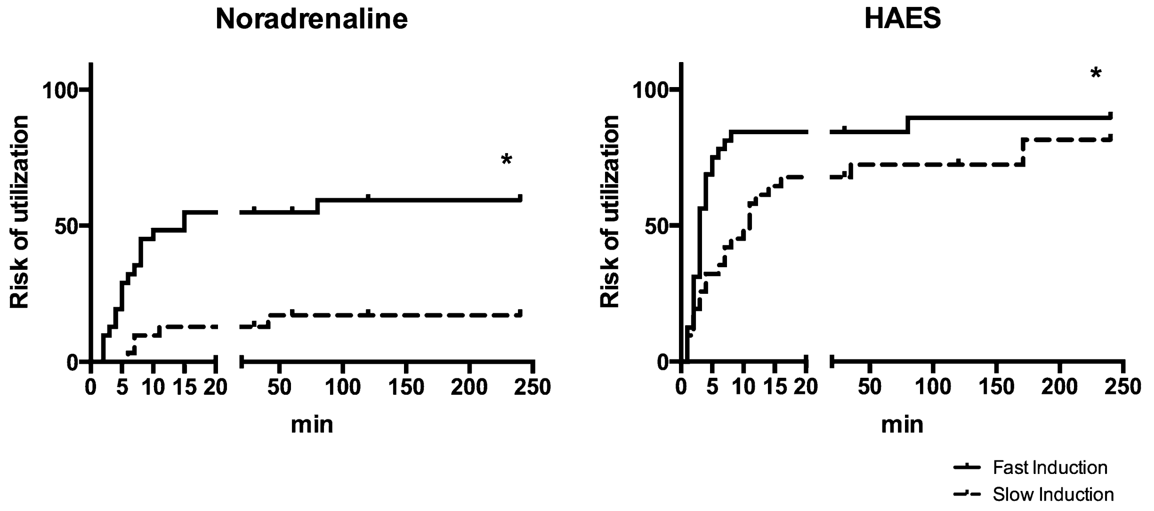

Fig. 2 Hazard curves for Noradrenaline and HAES utilization in in fast- (n = 32) and slow- (n = 35) inducted rats. *Asterisk* indicates p < 0.05

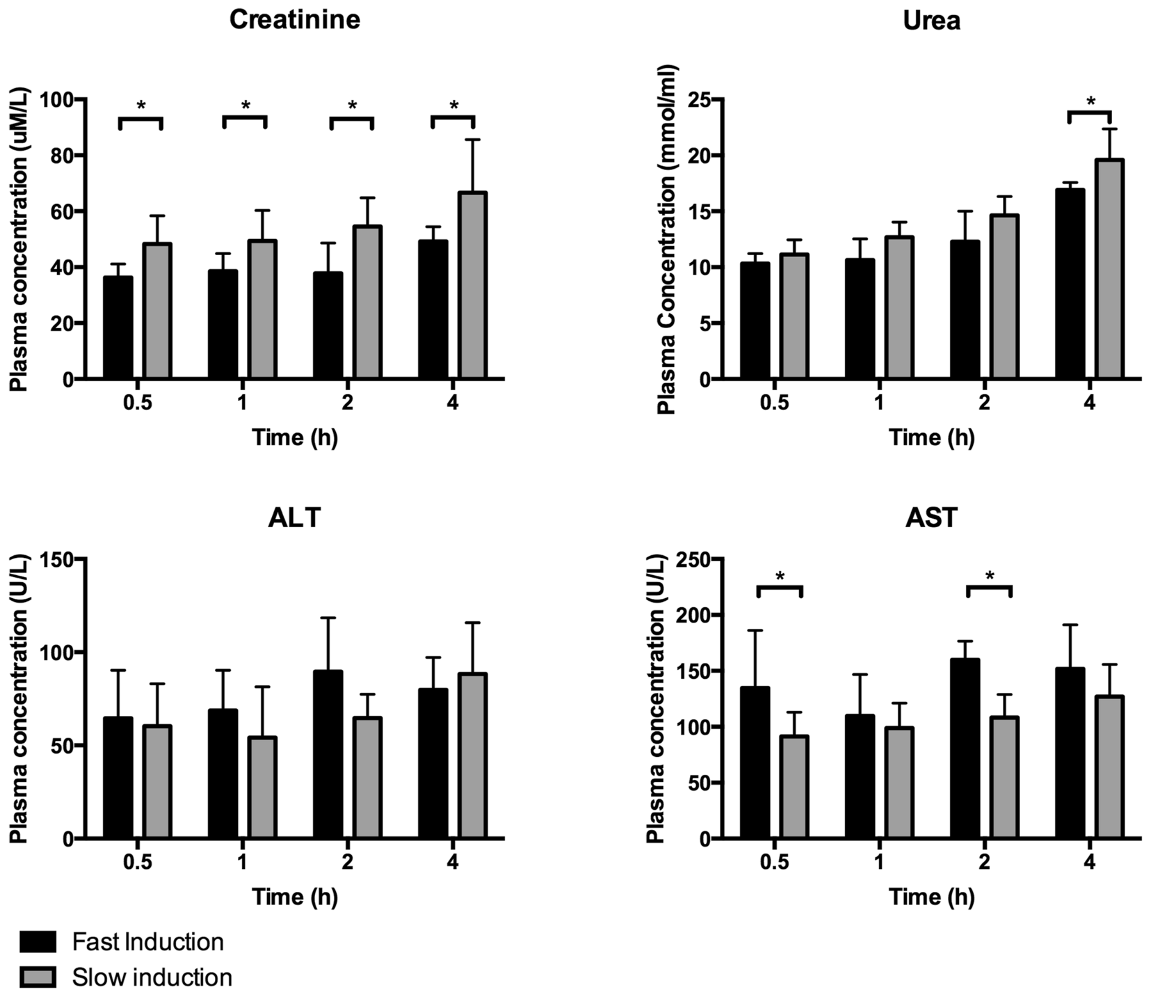

Fig. 3 Plasma levels of injury markers and function markers in fast inducted rats 0.5 (n = 8), 1 (n = 8), 2 (n = 6), and 4 h (n = 6) and slow inducted rats 0.5 (n = 8), 1 (n = 8), 2 (n = 7), and 4 h (n = 8) after BD. *Asterisk* indicates p < 0.05

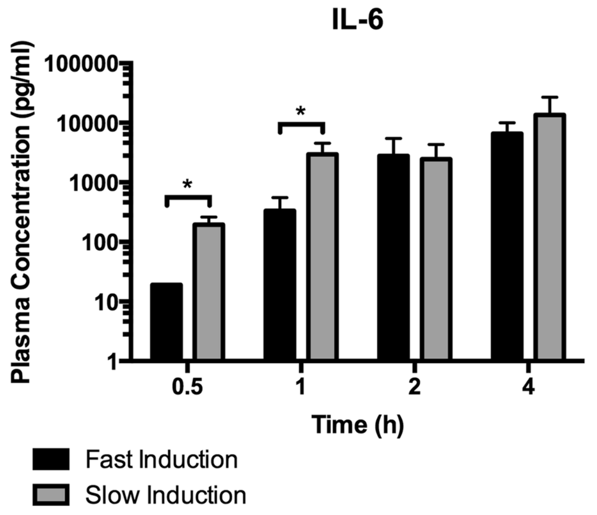

Fig. 4 IL-6 plasma levels in in fast inducted rats 0.5 (n = 8), 1 (n = 8), 2 (n = 6), and 4 h (n = 6) and slow inducted rats 0.5 (n = 8), 1 (n = 8), 2 (n = 7), and 4 h (n = 8) after BD. *Asterisk* indicates $p < 0.05$

increased due to slow induction compared to fast induction after 0.5 and 1 h of BD (p = 0.0014 and p = 0.0002, Fig. 4).

We assessed tissue inflammation by measuring the relative expression of pro-inflammatory genes in the kidney and liver. Relative TNF-α gene expression in the kidney showed no differences between fast and slow speed induction. In contrast, the relative IL-6 gene expression increased significantly due to slow induction compared to fast induction after 0.5 and 4 h (p = 0.0348 and p = 0.0270, Fig. 5a). Hepatic TNF-α gene expression increased significantly due to slow induction compared to fast induction after 4 h of BD (p = 0.0293). No difference was found in the relative gene expression of IL-6 between fast and slow induction (Fig. 5b). PMN infiltration in renal and hepatic tissue was assessed by His-48 staining. There was no difference in His-48 positive staining in the renal cortex and hepatic tissue between fast and slow induction (Fig. 6).

In order to study apoptotic pathways in renal and hepatic tissue, we measured the ratio between the relative Bax and Bcl-2 expression. No difference was found in the expression of this ratio in renal tissue between fast and slow induction. In contrast, the hepatic gene expression of the Bax/Bcl-2 ratio was significantly higher due to slow induction compared to fast induction after 4 h of BD (p = 0.0293, Fig. 7). Additionally, hepatic cleaved caspase-3 protein expression was significantly increased due to slow induction compared to fast induction after 4 h of BD (p = 0.001, Fig. 8).

Oxidative stress was assessed by measuring lipid peroxidation. MDA levels were significantly higher in renal tissue due to slow induction compared to fast induction after 4 h of BD (p = 0.01). Hepatic MDA levels were comparable between fast and slow induction groups after 4 h of BD (p = 0.48, Fig. 9).

Discussion

The speed at which brain insults progress to BD varies greatly in ICU patients. Even donors with the same nature of brain insults progress to BD at different speeds. After infarction of the middle cerebral artery, BD can typically manifest itself anywhere between 24 h and a week [18]. In a prospective study of patients with subarachnoid hemorrhage who progressed to BD, 26 % were still not declared BD after 1 week [19]. This large range in time intervals is the result of the complex pathophysiology of the processes leading to BD and reflective of the speed at which ICP increases [20]. As of yet, the speed at which ICP increases, has not been investigated as a possible determinant of renal and hepatic graft survival.

Here we report for the first time that the speed of BD induction affects functional, immunologic, apoptotic, and oxidative stress markers in the kidney and liver. In our experimental setting, we found that a slower speed of BD induction, elicits more detrimental renal and hepatic effects compared to a faster speed of BD induction. The effect of slower speed of BD induction is especially apparent in the kidney as renal function is diminished which was measured by serum creatinine values.

We showed that faster BD induction leads to more hemodynamic instability in the first hour after BD induction and therefore higher amounts of noradrenaline and HAES were required during this time period to maintain MAP within the physiological range. This is probably related to the higher peak of plasma catecholamine levels caused by fast BD induction as was shown by Shivalkar et al. [14]. Higher levels of catecholamines lead to increased myocardial load and injury. Myocardial injury causes a subsequent drop in blood pressure and increases the need of hemodynamic support [13]. The negative effect of fast speed induction on hemodynamic stability appears to fade over time as the administered amount of HAES and NA did not differ between fast and slow induction at 2 and 4 h of BD.

In our experiment, the effects of speed of BD induction on the kidney and liver become especially evident at later time-points of the BD period. Differences between both models with regard to the kidney are clearer since creatinine levels increase over time in both models but are significantly higher due to slow induction at all time points. The hypotensive phase observed during slow speed induction could explain the decreased renal function (prerenal acute kidney injury) at early BD time points which forms a "first hit" in slow-inducted rats. However,

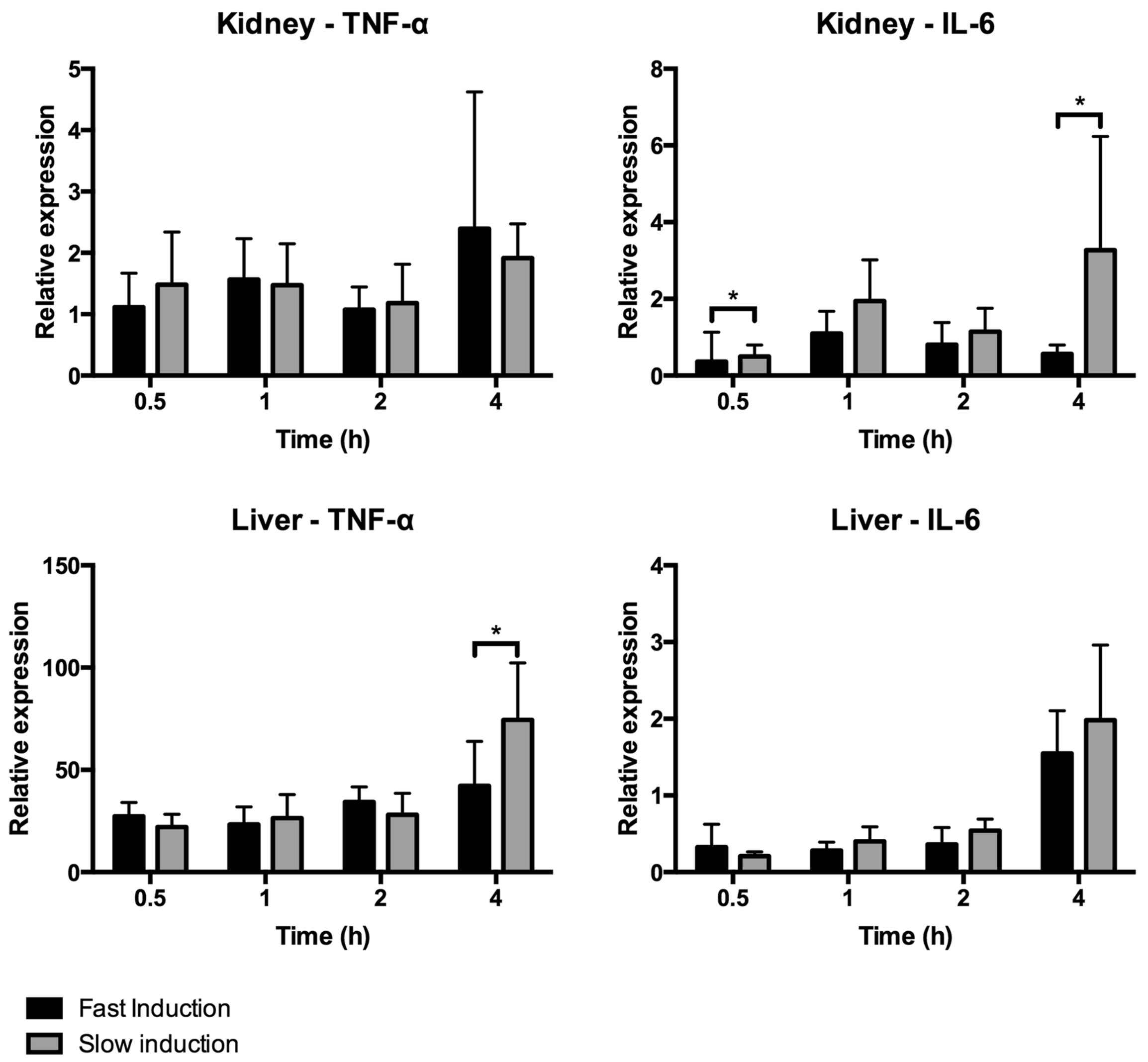

Fig. 5 Relative expression of inflammatory genes in renal and hepatic tissue in fast inducted rats 0.5 (n = 8), 1 (n = 8), 2 (n = 6), and 4 h (n = 6) and slow inducted rats 0.5 (n = 8), 1 (n = 8), 2 (n = 7), and 4 h (n = 8) after BD. The fold induction represents the relative expressions of these genes as compared to the expression level of the housekeeping GAPDH gene. *Asterisk* indicates $p < 0.05$

renal function shows no improvement over the course of the BD period even when MAP is maintained within the physiological range. Moreover, progressive diminished renal function was also observed after fast induction in which a hypotensive phase did not occur which indicates that local changes in the renal microcirculation are more likely to have caused the decreased renal function during the later stages of BD. We think that changes observed during the later stages of BD form a "second hit" in slow-inducted rats while this comprises the first (and single) hit in fast-inducted rats. Changes observed in the later stages of BD can be the result of one of the sole or combined effects of hemodynamic instability, inflammation, and hormonal impairment [7, 21, 22]. Furthermore, It is known that colloids like HAES can cause decreased renal function [23]. However, this could not explain the difference observed between groups, considering there was no difference in the administered amounts of HAES between fast- and slow-inducted rats. We therefore think that future studies should focus on the effect of speed of BD induction on graft function after transplantation. Possibly, strategies to ameliorate kidney function, such as machine perfusion, could be most advantageous in donors in which progression to BD occurs over a longer time period.

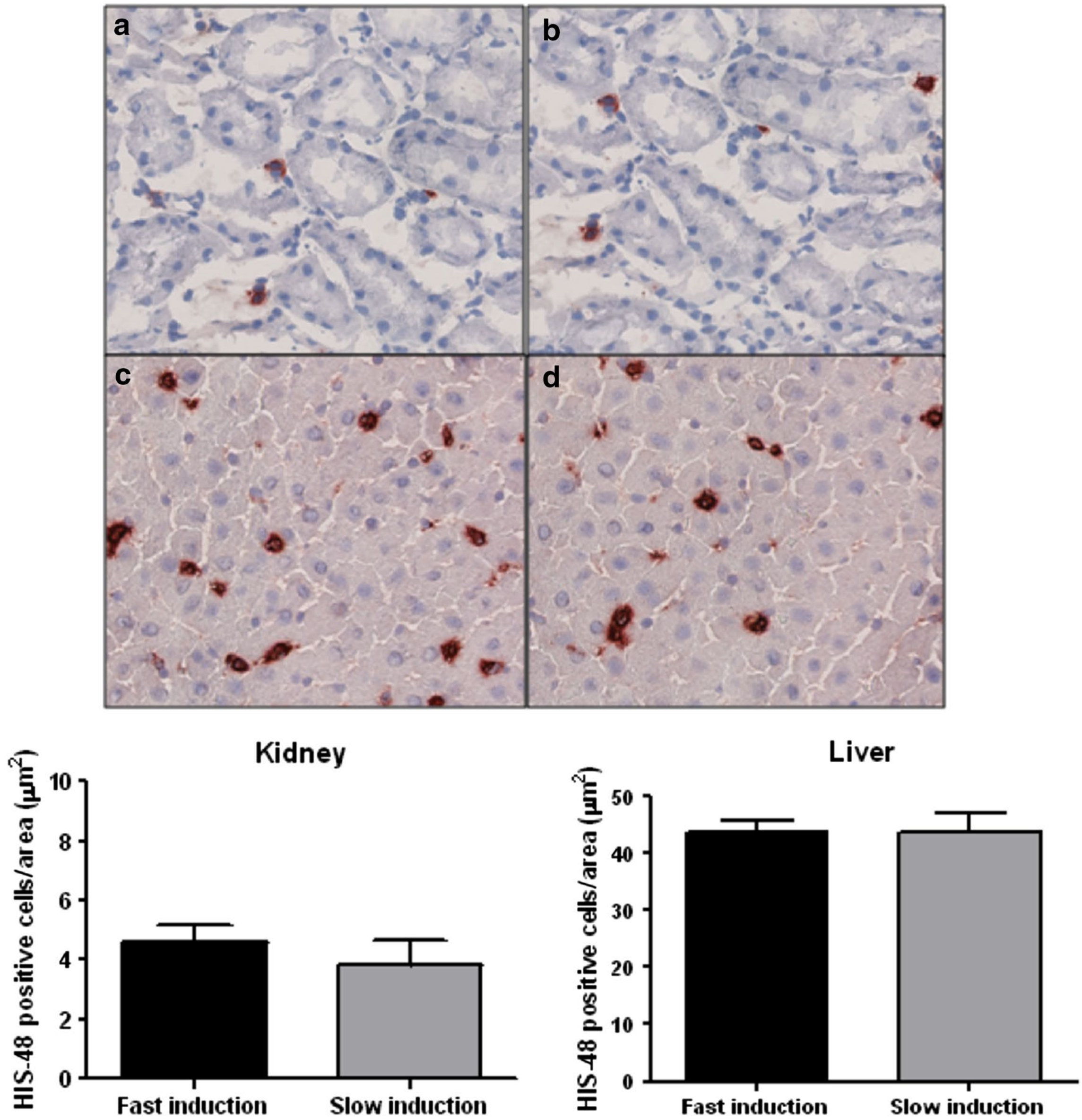

Fig. 6 Polymorphonuclear (PMN) influx in renal and hepatic tissue after 4 h of BD. Kidneys after **a** fast induction (n = 6) and **b** slow induction (n = 6) ×20 magnification factor. Livers after **c** fast induction (n = 6) and **d** slow induction (n = 6) ×10 magnification factor

Plasma ALT and AST levels are known to increase in brain-dead rats [6, 24]. In our experiment ALT levels, reflective of liver cell injury, showed no differences between both induction methods. However, AST levels were higher due to fast induction compared to slow induction after 0.5 and 2 h BD. We believe this not to be a reflection of liver damage due to no concurrent rise in ALT. Moreover, since AST is found in many tissues including the heart and lung, the early timepoints after which AST is increased, imply a causative role of the catecholamine storm and could be a reflection of lung and/or heart damage since these organs are affected by high levels of circulating catecholamines.

IL-6 is the cytokine most often implicated in brain death [25–28]. Plasma IL-6 levels increased significantly after slow induction compared to fast induction after 0.5 and 1 h of BD. This difference disappears over the course of the BD period which could be explained by the above

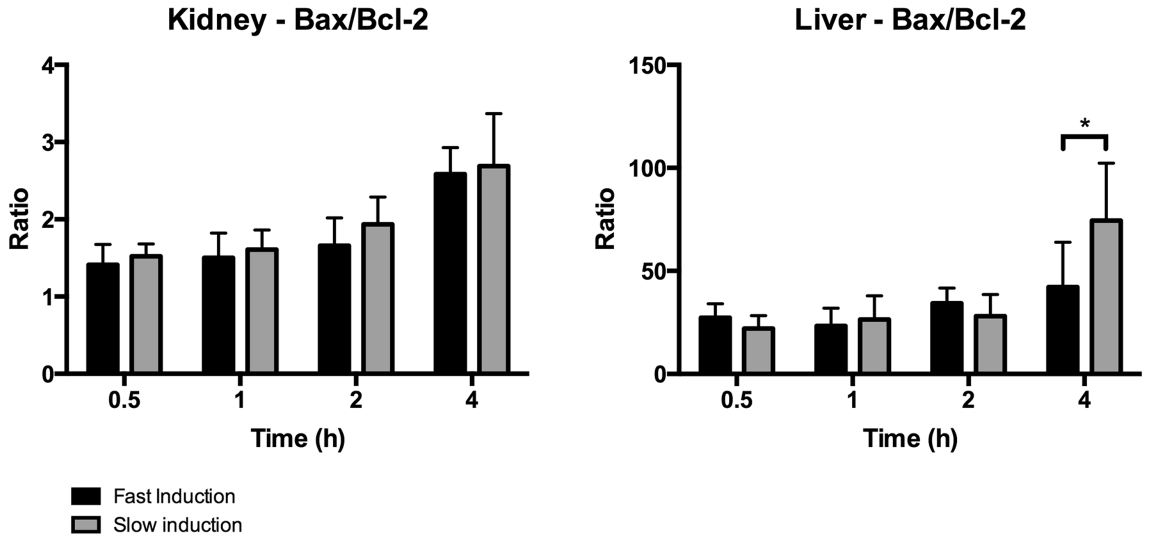

Fig. 7 Ratio of the relative BAX/Bcl-2 expressions in the kidney and liver in fast inducted rats 0.5 (n = 8), 1 (n = 8), 2 (n = 6), and 4 h (n = 6) and slow inducted rats 0.5 (n = 8), 1 (n = 8), 2 (n = 7), and 4 h (n = 8) after BD. The fold induction represents the relative expressions of these genes as compared to the expression level of the household GAPDH gene. *Asterisk* indicates $p < 0.05$

mentioned "first" and "second hit" in fast- and slow-inducted rats respectively. Therefore, systemic inflammatory markers like IL-6 might not be a good reflection of local inflammatory responses as these can be masked by the widespread events occurring in the brain dead donor.

The speed of BD induction leads to differences in immunologic and apoptotic responses in the kidney and liver. Renal IL-6 mRNA expression was significantly higher at 0.5 and 4 h BD due to slow BD induction which could be explained by the second hit theory as explained above. However, no differences in PMN influx were observed between both models which could be due to the fact that 4 h of BD was not sufficient to reveal a difference. Hepatic IL-6 levels did not differ between models and neither was there a difference in PMN influx. However, hepatic mRNA expression of TNF-α was significantly increased due to slow induction compared to fast induction after 4 h of BD. A concurrent significant increase was observed in the expression of caspase-3 due to slow induction. TNF-α is a known inducer of extrinsic apoptosis and therefore signalling through death receptor mediated pathways is plausible in our model [29]. Since TNF-α has a major implication in hepatic ischemia–reperfusion injury, hepatic TNF-α levels in human donors that progress to BD at different speeds should be assessed. [30–32]. Hepatic mRNA expression of the BAX/BcL-2 ratio was also significantly increased due to slow induction compared to fast induction which also suggests a possible role of intrinsic apoptosis through the permeabilization of mitochondria. The causal relationship of these processes and how they are initiated remains unclear and therefore, future investigations should focus on them in more depth. However, a possible cause could be the deposition of complement which has shown to occur in livers of brain-dead rats and which is a known inducer of apoptosis [24]. Renal mRNA expression of the BAX/BcL-2 ratio was not different between fast and slow BD induction. Moreover, there was no renal expression of caspase-3 after both fast and slow induction. This could indicate that the renal insults caused by BD are not severe enough to initiate programmed cell death or that other forms of cell death are initiated which we did not study.

The formation of oxidative stress in brain dead kidneys has been well documented [25, 33]. Lipid peroxidation, an oxidative stress marker, in brain dead donors is an independent risk factor for renal graft dysfunction in transplant recipients [34]. We report here that slower BD induction leads to more renal lipid peroxidation compared to fast induction. We believe that possible changes in the renal microcirculation could be related to the observed differences in lipid peroxidation. In the context of acute kidney injury, oxidative processes mediate peritubular microcirculatory changes which lead to diminished renal perfusion and function [35, 36]. However, causal roles between microcirculatory changes and lipid peroxidation cannot be inferred from this study. No difference was observed in hepatic lipid peroxidation

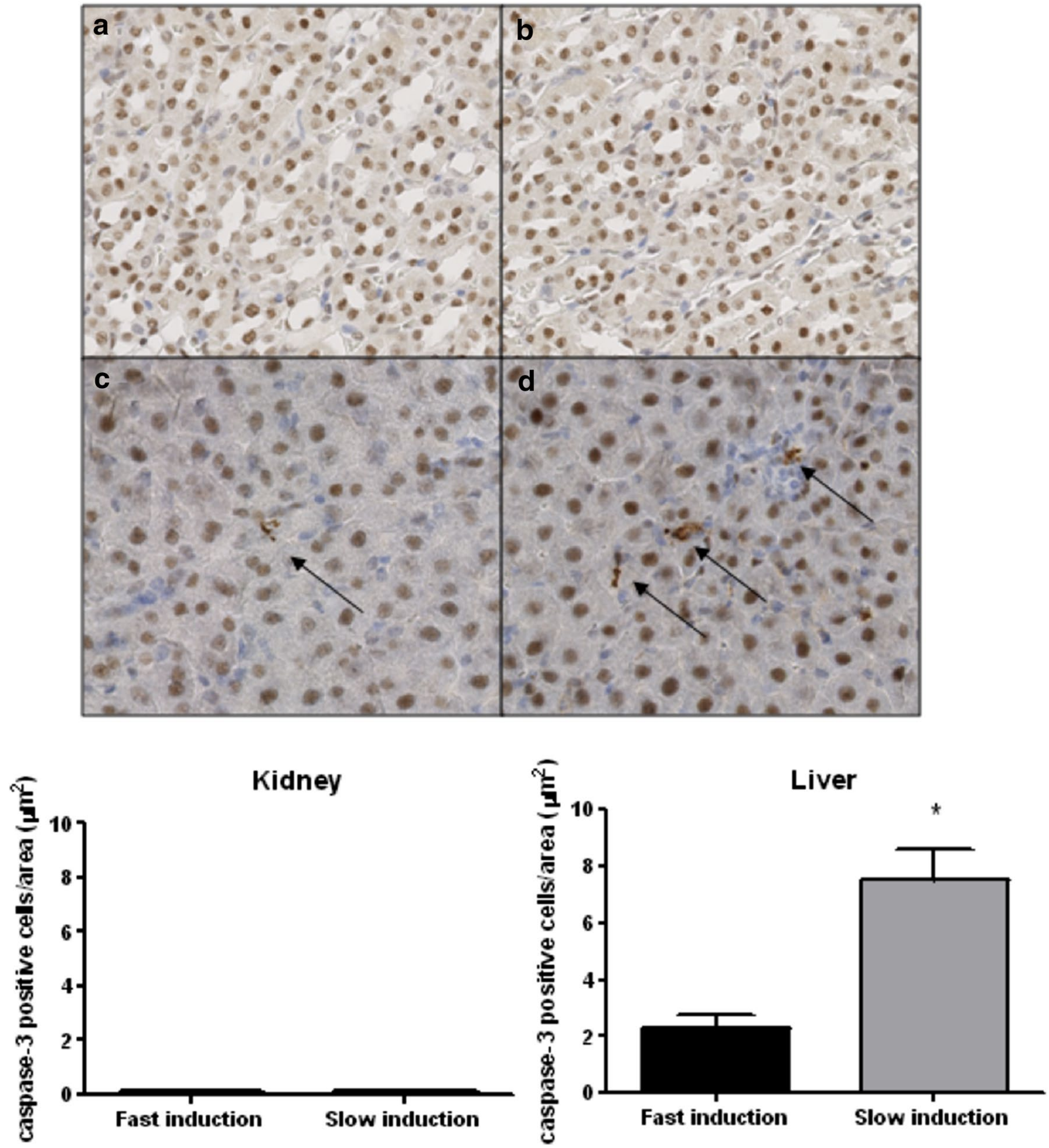

Fig. 8 Cleaved-caspase 3 expression in renal and hepatic tissue after 4 h of BD. Kidneys after **a** fast induction (n = 6) and **b** slow induction (n = 8) ×10 magnification factor. Livers after **c** fast induction (n = 6) and **d** slow induction (n = 6) ×10 magnification factor. *Asterisk* indicates $p < 0.01$

between fast and slow BD induction which implies that oxidative stress does not influence other hepatic effects we observed.

Conclusion

The presented data provide an initial broad overview of changes elicited by the speed of BD induction. We found that a slower speed of BD induction leads to more detrimental effects in the kidney and liver. This could indicate that speed of BD induction should be taken into account when decisions about organ allocation are made. The effects of speed of BD induction are more pronounced in the kidney as renal function is diminished more due to a slower speed. Nevertheless, hepatic inflammatory and apoptotic markers are increased more due to slow induction. We believe that increased

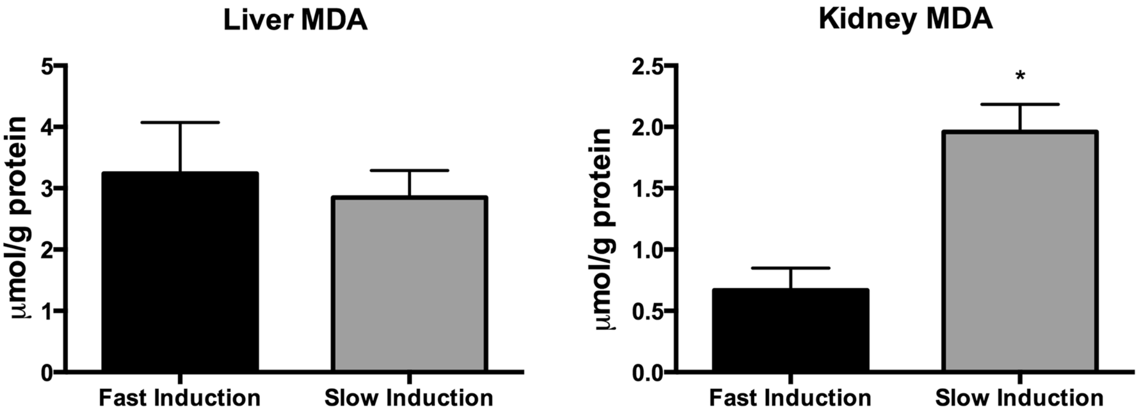

Fig. 9 MDA levels in renal and hepatic tissue from in fast- (n = 6)and slow- (n = 8) inducted rats after 4 h of BD. *Asterisk* indicates $p < 0.05$

knowledge about the processes leading up to BD can be of valuable use for brain-dead donor management and thereby improve transplantation outcomes.

Abbreviations

ADH: antidiuretic hormone; ALT: alanine transaminase; AST: aspartate transaminase; Bax: bcl-2-associated X protein; Bcl-2: B-cell lymphoma 2; BD: brain death; BSA: bovine serum albumin; cC3: cleaved caspase 3; GAPDH: glyceraldehyde 3-phosphate dehydrogenase; ICP: intracranial pressure; HAES: polyhydroxyethyl starch; HO-1: heme oxygenase 1; I/R-injury: ischemia/reperfusion-injury; MAP: mean arterial pressure; MCP-1: monocyte chemotactic protein 1; MDA: malondialdehyde; NA: noradrenaline; ROS: reactive oxygen species; TNF-α: tumor necrosis factor-alpha; T_3: 3,3′,5-triiodo-l-thyronine; T_4: thyroxine.

Authors' contributions

RR, HD, LH and HC designed the experiments. RR, HD, HC, WJ and BY performed the experiments. RR, HD and LH analyzed data and wrote the paper. All authors read and approved the final manuscript.

Author details

[1] Department of Surgery, University Medical Center Groningen, University of Groningen, CMC V, Y2144, Hanzeplein 1, 9713 GZ, Groningen, The Netherlands. [2] Physiopathology Program, Institute of Biomedical Sciences, Faculty of Medicine, University of Chile, Santiago, Chile. [3] Department of Cardiothoracic Surgery, University Medical Center Groningen, Groningen, The Netherlands.

Competing interests

The authors declare that they have no competing interests.

Received: 17 February 2016 Accepted: 29 April 2016
Published online: 19 May 2016

References

1. Mizraji R, Alvarez I, Palacios RI, Fajardo C, Berrios C, Morales F, et al. Organ donation in Latin America. Transplant Proc. 2007;39(2):333–5.
2. Sung RS, Galloway J, Tuttle-Newhall JE, Mone T, Laeng R, Freise CE, et al. Organ donation and utilization in the United States, 1997–2006. Am J Transplant. 2008;8(4 Pt 2):922–34.
3. Terasaki PI, Cecka JM, Gjertson DW, Takemoto S. High survival rates of kidney transplants from spousal and living unrelated donors. N Engl J Med. 1995;333(6):333–6.
4. Feng S, Goodrich NP, Bragg-Gresham JL, Dykstra DM, Punch JD, DebRoy MA, et al. Characteristics associated with liver graft failure: the concept of a donor risk index. Am J Transplant. 2006;6(4):783–90.
5. Pessione F, Cohen S, Durand D, Hourmant M, Kessler M, Legendre C, et al. Multivariate analysis of donor risk factors for graft survival in kidney transplantation. Transplantation. 2003;75(3):361–7.
6. Weiss S, Kotsch K, Francuski M, Reutzel-Selke A, Mantouvalou L, Klemz R, et al. Brain death activates donor organs and is associated with a worse I/R injury after liver transplantation. Am J Transplant. 2007;7(6):1584–93.
7. Nijboer WN, Schuurs TA, Van Der Hoeven JAB, Fekken S, Wiersema-Buist J, Leuvenink HGD, et al. Effect of brain death on gene expression and tissue activation in human donor kidneys. Transplantation. 2004;78(7):978–86.
8. Murugan R, Venkataraman R, Wahed AS, Elder M, Hergenroeder G, Carter M, et al. Increased plasma interleukin-6 in donors is associated with lower recipient hospital-free survival after cadaveric organ transplantation*. Crit Care Med. 2008;36(6):1810–6.
9. Jassem W, Koo DDH, Cerundolo L, Rela M, Heaton ND, Fuggle SV. Leukocyte infiltration and inflammatory antigen expression in cadaveric and living-donor livers before transplant. Transplantation. 2003;75(12):2001–7.
10. Nijboer WN, Schuurs TA, van der Hoeven JAB, Leuvenink HGD, van der Heide JJH, van Goor H, et al. Effects of brain death on stress and inflammatory response in the human donor kidney. Transplant Proc. 2005;37(1):367–9.
11. Novitzky D, Cooper DKC, Rosendale JD, Kauffman HM. Hormonal therapy of the brain-dead organ donor: experimental and clinical studies. Transplantation. 2006;82(11):1396–401.
12. Novitzky D, Horak A, Cooper DK, Rose AG. Electrocardiographic and histopathologic changes developing during experimental brain death in the baboon. TPS. 1989;21(1 Pt 3):2567–9.
13. Cooper DK, Novitzky D, Wicomb WN. The pathophysiological effects of brain death on potential donor organs, with particular reference to the heart. Ann R Coll Surg Engl. 1989;71(4):261–6.
14. Shivalkar B, Van Loon J, Wieland W, Tjandra-Maga TB, Borgers M, Plets C, et al. Variable effects of explosive or gradual increase of intracranial pressure on myocardial structure and function. Circulation. 1993;87(1):230–9.
15. Cohen O, La Zerda De DJ, Beygui R, Hekmat D, Laks H. Donor brain death mechanisms and outcomes after heart transplantation. TPS. 2007;39(10):2964–9.
16. Feng S, Goodrich NP, Bragg-Gresham JL, Dykstra DM, Punch JD, DebRoy MA, et al. Characteristics associated with liver graft failure: the concept of a donor risk index. Am J Transplant. 2006;6(4):783–90.
17. Cuende N, Miranda B, Cañón JF, Garrido G, Matesanz R. Donor characteristics associated with liver graft survival. Transplantation. 2005;79(10):1445–52.
18. Hacke W, Schwab S, Horn M, Spranger M, De Georgia M, von Kummer R. "Malignant" middle cerebral artery territory infarction: clinical course and prognostic signs. Arch Neurol. 1996;53(4):309–15.

19. Lantigua H, Ortega-Gutierrez S, Schmidt JM, Lee K, Badjatia N, Agarwal S, et al. Subarachnoid hemorrhage: who dies, and why? Crit Care. 2015;19(1):309.
20. Simard JM, Kent TA, Chen M, Tarasov KV, Gerzanich V. Brain oedema in focal ischaemia: molecular pathophysiology and theoretical implications. Lancet Neurol. 2007;6(3):258–68.
21. Van Der Hoeven JAB, Molema G, Ter Horst GJ, Freund RL, Wiersema J, van Schilfgaarde R, et al. Relationship between duration of brain death and hemodynamic (in)stability on progressive dysfunction and increased immunologic activation of donor kidneys. Kidney Int. 2003;64(5):1874–82.
22. Novitzky D, Cooper DK, Morrell D, Isaacs S. Change from aerobic to anaerobic metabolism after brain death, and reversal following triiodothyronine therapy. Transplantation. 1988;45(1):32–6.
23. Myburgh JA, Mythen MG. Resuscitation fluids. N Engl J Med. 2013;369(13):1243–51.
24. Rebolledo R, Liu B, Akhtar MZ, Ottens PJ, Zhang J-N, Ploeg RJ, et al. Prednisolone has a positive effect on the kidney but not on the liver of brain dead rats: a potencial role in complement activation. J Transl Med. 2014;12(1):111.
25. Schuurs TA, Morariu AM, Ottens PJ, Hart NAT, Popma SH, Leuvenink HGD, et al. Time-dependent changes in donor brain death related processes. Am J Transplant. 2006;6(12):2903–11.
26. Koudstaal LG, t Hart NA, Ottens PJ, Van Den Berg A, Ploeg RJ, Van Goor H, et al. Brain death induces inflammation in the donor intestine. Transplantation. 2008;86(1):148–54.
27. Saat TC, Susa D, Roest HP, Kok NFM, van den Engel S, IJzermans JNM, et al. A comparison of inflammatory, cytoprotective and injury gene expression profiles in kidneys from brain death and cardiac death donors. Transplantation. 2014;98(1):15–21.
28. Schuurs TA, Gerbens F, Van Der Hoeven JAB, Ottens PJ, Kooi KA, Leuvenink HGD, et al. Distinct transcriptional changes in donor kidneys upon brain death induction in rats: insights in the processes of brain death. Am J Transplant. 2004;4(12):1972–81.
29. Van Der Hoeven JAB, Moshage H, Schuurs T, Nijboer M, van Schilfgaarde R, Ploeg RJ. Brain death induces apoptosis in donor liver of the rat. Transplantation. 2003;76(8):1150–4.
30. Spencer NY, Zhou W, Li Q, Zhang Y, Luo M, Yan Z, et al. Hepatocytes produce TNF-α following hypoxia-reoxygenation and liver ischemia-reperfusion in a NADPH oxidase- and c-Src-dependent manner. AJP: gastrointestinal and liver. Physiology. 2013;305(1):G84–94.
31. Goto M, Takei Y, Kawano S, Tsuji S, Fukui H, Fushimi H, et al. Tumor necrosis factor and endotoxin in the pathogenesis of liver and pulmonary injuries after orthotopic liver transplantation in the rat. Hepatology. 1992;16(2):487–93.
32. Ben-Ari Z, Hochhauser E, Burstein I, Papo O, Kaganovsky E, Krasnov T, et al. Role of anti-tumor necrosis factor-alpha in ischemia/reperfusion injury in isolated rat liver in a blood-free environment. Transplantation. 2002;73(12):1875–80.
33. Morariu AM, Schuurs TA, Leuvenink HGD, van Oeveren W, Rakhorst G, Ploeg RJ. Early events in kidney donation: progression of endothelial activation, oxidative stress and tubular injury after brain death. Am J Transplant. 2008;8(5):933–41.
34. Kosieradzki M, Kuczynska J, Piwowarska J, Wegrowicz-Rebandel I, Kwiatkowski A, Lisik W, et al. Prognostic significance of free radicals: mediated injury occurring in the kidney donor. Transplantation. 2003;75(8):1221–7.
35. Seely KA, Holthoff JH, Burns ST, Wang Z, Thakali KM, Gokden N, et al. Hemodynamic changes in the kidney in a pediatric rat model of sepsis-induced acute kidney injury. Am J Physiol Renal Physiol. 2011;301(1):F209–17.
36. Wang Z, Holthoff JH, Seely KA, Pathak E, Spencer HJ, Gokden N, et al. Development of oxidative stress in the peritubular capillary microenvironment mediates sepsis-induced renal microcirculatory failure and acute kidney injury. Am J Pathol. 2012;180(2):505–16.

Submit your next manuscript to BioMed Central and we will help you at every step:

- We accept pre-submission inquiries
- Our selector tool helps you to find the most relevant journal
- We provide round the clock customer support
- Convenient online submission
- Thorough peer review
- Inclusion in PubMed and all major indexing services
- Maximum visibility for your research

Submit your manuscript at www.biomedcentral.com/submit

BioMed Central
